# Adherence to Disease Modifying Drugs among Patients with Multiple Sclerosis in Germany: A Retrospective Cohort Study

**DOI:** 10.1371/journal.pone.0133279

**Published:** 2015-07-27

**Authors:** Kerstin Hansen, Katrin Schüssel, Marita Kieble, Johanna Werning, Martin Schulz, Robert Friis, Dieter Pöhlau, Norbert Schmitz, Joachim Kugler

**Affiliations:** 1 TU Dresden, Medizinische Fakultät, Lehrstuhl für Gesundheitswissenschaften / Public Health, Dresden, Germany; 2 Deutsches Arzneiprüfungsinstitut e. V. (DAPI), Berlin, Germany; 3 Department of Health Science, California State University, Long Beach, California, United States of America; 4 Kamillus-Klinik, Asbach, Germany; 5 Douglas Mental Health University Institute, FBC Building, Montreal, Canada; 6 TU Dresden, Medizinische Fakultät, Bereich Allgemeinmedizin, Dresden, Germany; University of Oxford, UNITED KINGDOM

## Abstract

**Background:**

Long-term therapies such as disease modifying therapy for Multiple Sclerosis (MS) demand high levels of medication adherence in order to reach acceptable outcomes. The objective of this study was to describe adherence to four disease modifying drugs (DMDs) among statutorily insured patients within two years following treatment initiation. These drugs were interferon beta-1a i.m. (Avonex), interferon beta-1a s.c. (Rebif), interferon beta-1b s.c. (Betaferon) and glatiramer acetate s.c. (Copaxone).

**Methods:**

This retrospective cohort study used pharmacy claims data from the data warehouse of the German Institute for Drug Use Evaluation (DAPI) from 2001 through 2009. New or renewed DMD prescriptions in the years 2002 to 2006 were identified and adherence was estimated during 730 days of follow-up by analyzing the medication possession ratio (MPR) as proxy for compliance and persistence defined as number of days from initiation of DMD therapy until discontinuation or interruption.

**Findings:**

A total of 52,516 medication profiles or therapy cycles (11,891 Avonex, 14,060 Betaferon, 12,353 Copaxone and 14,212 Rebif) from 50,057 patients were included into the analysis. Among the 4 cohorts, no clinically relevant differences were found in available covariates. The Medication Possession Ratio (MPR) measured overall compliance, which was 39.9% with a threshold MPR≥0.8. There were small differences in the proportion of therapy cycles during which a patient was compliant for the following medications: Avonex (42.8%), Betaferon (40.6%), Rebif (39.2%), and Copaxone (37%). Overall persistence was 32.3% at the end of the 24 months observation period, i.e. during only one third of all included therapy cycles patients did not discontinue or interrupt DMD therapy. There were also small differences in the proportion of therapy cycles during which a patient was persistent as follows: Avonex (34.2%), Betaferon (33.4%), Rebif (31.7%) and Copaxone (29.8%).

**Conclusions:**

Two years after initiating MS-modifying therapy, only 30–40% of patients were adherent to DMDs.

## Introduction

Multiple sclerosis (MS) is an inflammatory, chronic, and degenerative autoimmune disease of the central nervous system that, at present, cannot be cured. Approximately 140,000 Germans suffer from MS [1]. Since 2001 four immune-modulatory-drugs (IMDs), also referred to as disease modifying-drugs (DMDs), have been approved in Germany for relapsing remitting multiple sclerosis (RRMS): interferon (INF) beta-1a i.m. (Avonex), administered once a week, interferon (INF) beta-1a s.c. (Rebif), administered three times a week, interferon beta-1b s.c. (Betaferon), administered every second day, and glatiramer acetate s.c. (Copaxone), administered daily. DMDs should reduce the number and frequency of exacerbations and slow down the progression of cognitive and physical disability. All of these DMDs require regular, long-term administration by injection, but the route of administration (subcutaneous s.c. or intramuscular i.m.), and frequency of administration differ. Guidelines of neurologist experts recommend a treatment over a minimum of two years, with the benefit expected after 2 years of continuous treatment [2, 3]. To achieve maximum treatment efficacy for chronic diseases, high long-term adherence is necessary. The World Health Organization (WHO) defines treatment adherence as both compliance and persistence. Compliance means the correct application of a prescribed therapy regarding dose, schedule and method of application, while persistence refers to the entire recommended treatment period [4]. In international studies, depending on the method of measurement, between 15% and 53% of patients undergoing DMD treatment became non-adherent within the first two years [3,5–25]. Patients with adherence to DMD therapy of less than 24 months will probably produce high costs (in Germany approximately 1,500€/month / person) without benefit.

In this study we included the records of more than 80% of patients who were covered by Germany´s statutory health insurance scheme and who obtained a DMD during the period of 2002 through 2006. We analyzed adherence by two indirect measurement methods. First, we analyzed the medication possession ratio (MPR) as proxy for compliance. Second, we assessed persistence (continuity of therapy) by determining whether there was a termination of treatment or a defined gap in a patient´s medication profile. Furthermore, we examined medication adherence for the four groups, characterized by the DMD the patient received initially, i.e. interferon (INF) beta-1a i.m. (Avonex), interferon (INF) beta-1a s.c. (Rebif), interferon (INF) beta-1b s.c. (Betaferon), and glatiramer acetate s.c. (Copaxone).

## Methods

### Database

The German Institute for Drug Use Evaluation (Deutsches Arzneiprüfungsinstitut e. V. (DAPI)) is a non-profit association active in the area of pharmacoeconomics and pharmacoepidemiology. The DAPI database contains pharmacy claims data of patients insured by the statutory health insurance system from more than 80% of German community pharmacies. In Germany, about 85% of the population (70.2 million patients) is insured by the statutory health insurance program [26]. This retrospective cohort study focused on data for the period January 1, 2001, through December 31, 2009. The prescription data of the DAPI database are linked to the ABDA database which contains a complete inventory of German medical products [27].

The drug claims data within the DAPI database include a unique product identification code (Pharmazentralnummer, PZN), which allows linkage with information on pharmaceutical ingredients, strength, form of administration and quantity required for calculation of days supplied with medication. Additional information includes the date of prescription, the medical specialist group, the region of the prescribing physician, and the insurance status of the patient (full member, family member, retiree). Other personal data such as name, address, age or gender are not included. A de-identified patient code allows for follow-up of a patient´s prescriptions over several years. Not included in the database are prescriptions funded by private health insurance funds, medical samples obtained directly from physicians, medication provided in hospitals, and medication obtained by mail order from pharmacies located abroad. In the database there is no information about demographics such as age and sex, or clinical information about diagnosis, hospitalization, or laboratory tests.

The study was performed according to the recommendations for Good Practice for Secondary Data analysis [28]. The study was approved by the ethic committee of the Dresden Medical School and it was registered at the European Network of Centres for Pharmacoepidemiology and Pharmacovigilance (ENCePP) under the acronym ‘ADAPIMS’ in 2013.

### Cohort Definition

**Fig 1:**
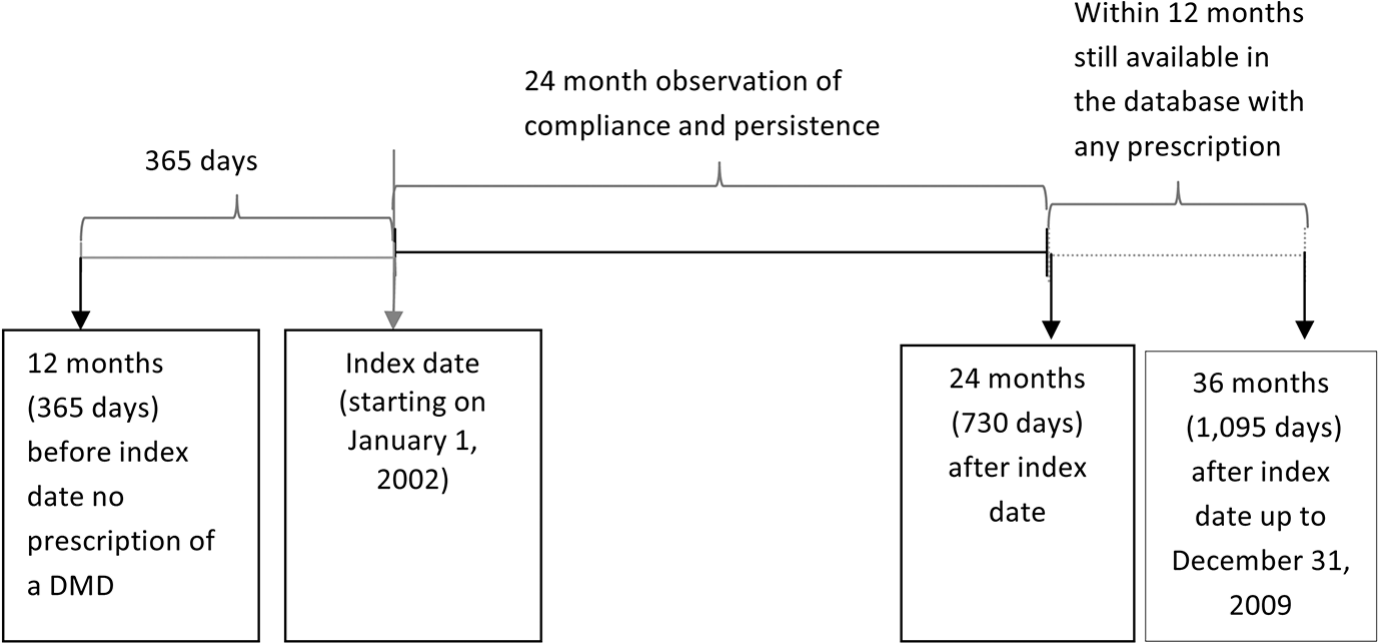
Cohort definition and observation period of the study.

Incident users of DMDs were identified with their first (= index) prescription of any DMD during the period between January 01, 2002, and December 31, 2006. Incident use was defined by not having received DMDs up to 365 days prior to the index prescription. Within the long total observation period it is possible to find more than one medication profile (therapy cycle) for the same patient, since a patient could restart DMD therapy after a treatment interruption of more than 365 days. A profile was allocated to one of the four study groups depending on the type of DMD of the index prescription. In order to ensure completeness of follow-up for at least 24 months, patients were excluded they did not appear in the database with any prescription within 24 to 36 months after the index date. Thus, patients who may have changed healthcare companies or may have died were not incorrectly classified as non-compliant. Furthermore, implausible medication profiles with more than 40 different ATC codes prescribed during 180 days before the index date were excluded from analysis, assuming that data processing errors (mostly during electronic transformation from paper-based prescriptions) may have caused this implausibility (Fig 1).

There was a 12 months pre-observation period, followed by a 24 months observation period and a 12 months follow-up period. Because of the long total observation period of 8 years, it would be possible to find more than one medication profile for the same patient (up to four profiles or cycles). For example, this situation could happen when early in the observation period (for example, during 2002) a patient had only one DMD prescription, then stopped therapy and after another 12-months interval started with another DMD, which created a new and second medication profile. Multiple profiles were found for 4.7% of the patients in the main cohort; the remaining 95.3% of the patients had only one medication profile. In a sensitivity analysis, we included only one medication profile per person.

### Measuring adherence

To estimate adherence to prescribed medications after initiation of DMDs, we measured medication compliance by using the medication possession ratio (MPR). Medication persistence was measured by determining the time from initiation (index date) to discontinuation of therapy or to a defined gap in a patient´s medication profile [29].

#### Medication Possession Ratio (MPR)

In general, the MPR is calculated from the number of doses dispensed in relation to the dispensing period of time reported as percentages [29]. In our study the denominator represented the observation period, which was a fixed duration defined as 730 days (24 months). The numerator consisted of the number of days in which the patient was covered by a prescribed DMD medication, irrespective of the type of DMD. Every pre-filled syringe of a DMD was counted as a single dose. The daily dose was calculated according to the dosage schedule provided by the Summary of Product Characteristics (SPC). If subsequent prescription was provided before a patient´s medication had been depleted, this occurrence was considered to be stockpiling the medication. Stockpiling from the previous, but not from earlier prescriptions was taken into account in the calculation of MPR and persistence. Sufficient compliance was considered for medication profiles showing a MPR≥0.8. In the sensitivity analyses, we analyzed both MPR≥0.7 and MPR≥0.9 as cut-off.

#### Persistence

We measured persistence as the number of days from DMD initiation (index date) to the occurrence of a first gap of more than one-fold duration of medication supply of the previous prescription in the medication profile. For example, if a prescription covered medication supply for 84 days, the medication profile was defined as interrupted if there was no repeat prescription within 84+84 = 168 days (84 day duration of medication supply plus 84 days allowable gap interval).

A patient was considered to be persistent for the respective medication profile if there was no medication gap greater than that described above.

#### Statistical analyses

For cohort characteristics proportions for categorical variables and mean values for continuous variables were determined. For compliance mean (+- standard deviation), median MPR and proportions of therapy cycles during which a patient was compliant were calculated. Box plots are presented to examine the distribution of MPRs within the study groups. Proportions of therapy cycles during which a patient was persistent were computed and persistence probabilities were visualized using Kaplan-Meier curves. All statistical analyses were performed using SPSS.

## Results

### Patient characteristics

**Fig 2:**
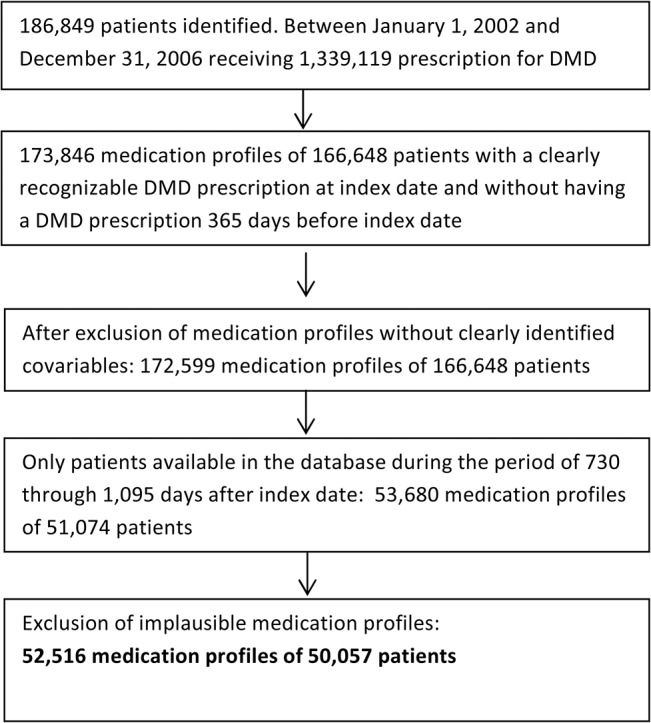
Flow chart of inclusion and exclusion criteria and remaining medication profiles/patients.

Prescriptions were available in the database for a total of 186,849 patients with a DMD for the period of January 01, 2002, through December 31, 2006. We excluded patient profiles that had no clearly recognizable DMD prescription at the index date (i.e. simultaneous prescription of two different DMDs), or profiles that had a DMD pre-treatment during 365 days before the index date (i.e. prevalent DMD user). In addition, we excluded profiles with unclear covariables at the index date and profiles with incomplete follow-up-information, i.e. that were not available in the database from 730 days (24 months) through 1,095 days (36 months) after the index date. A total of 52,516 medication profiles from 50,057 patients were included in the analyses (Fig 2).

Table 1 gives an overview over variables such as prescribing medical specialist, region, status of insurance, index year, pretreatment with anti-depressants and muscle relaxants, and number of ATC-codes of pretreatment for the 4 cohorts who used the 4 DMDs Avonex, Betaferon, Copaxone or Rebif (Table 1).

**Table 1:** Cohort characteristics of the main study cohort at the index date.

Characteristics		Overall	Avonex	Betaferon	Copaxone	Rebif
DMD (%)		52516 (100.0)	11891 (22.6)	14060 (26.8)	12353 (23.5)	14212 (27.1)
Medical specialist (%)	Neurologist	42602 (81.1)	9845 (82.8)	11532 (82.0)	10207 (82.6)	11018 (77.5)
	General practitioner	5236 (10.0)	1148 (9.7)	1382 (9.8)	1017 (8.2)	1689 (11.9)
	Institutions	2615 (5.0)	420 (3.5)	618 (4.4)	688 (5.6)	889 (6.3)
	Others/not specified	2063 (3.9)	478 (4.0)	528 (3.8)	441 (3.6)	616 (4.3)
Region (%)	West	42016 (80.0)	9491 (79.8)	11145 (79.3)	10042 (81.3)	11338 (79.8)
	East	6379 (12.1)	1469 (12.4)	1984 (14.1)	1159 (9.4)	1767 (12.4)
	Major cities (Berlin, Hamburg, Bremen)	4121 (7.8)	931 (7.8)	931 (6.6)	1152 (9.3)	1107 (7.8)
Status of insurance (%)	member	34645 (66.0)	8017 (67.4)	8742 (62.2)	8224 (66.6)	9662 (68.0)
	dependents' co-insurance	10819 (20.6)	2237 (18.8)	3471 (24.7)	2545 (20.6)	2566 (18.1)
	retiree	7052 (13.4)	1637 (13.8)	1847 (13.1)	1584 (12.8)	1984 (14.0)
Index-year (%)	2002	9458 (18.0)	1723 (14.5)	2600 (18.0)	2412 (19.5)	2723 (19.2)
	2003	10127 (19.3)	2254 (19.0)	2821 (20.1)	2260 (18.3)	2792 (19.6)
	2004	10120 (19.3)	2504 (21.1)	1684 (19.1)	2273 (18.4)	2659 (18.7)
	2005	11683 (22.2)	2882 (24.2)	3084 (21.9)	2684 (21.7)	3033 (21.3)
	2006	11128 (21.2)	2528 (21.3)	2871 (20.4)	2724 (22.1)	3005 (21.1)
Antidepressants dispensed (%)		11487 (21.9)	2493 (21.0)	3138 (22.3)	2989 (24.2)	2867 (20.2)
Muscle-relaxants dispensed (%)		7018 (13.4)	1460 (12.3)	2162 (15.4)	1674 (13.6)	1722 (12.1)
Number of ATC-codes (pretreatment) Average (standard deviation, SD)		8.32 (±8.30)	8.28 (±8.37)	8.71 (±8.56)	8.13 (±8.01)	8.14 (±8.22)

### Compliance/ Medication Possession ratio

**Fig 3:**
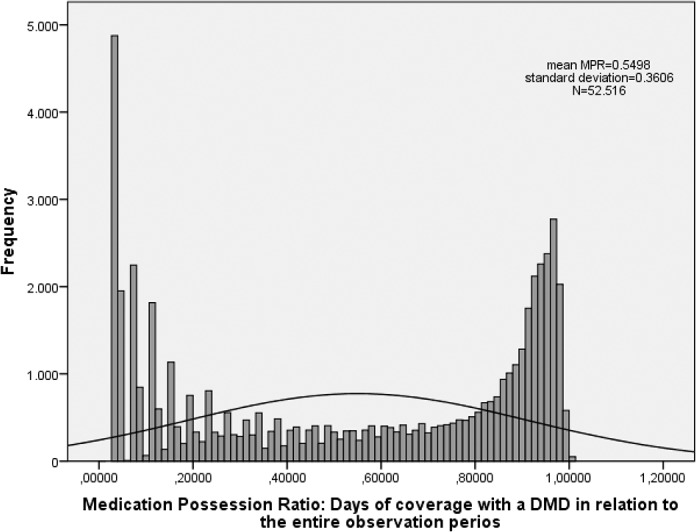
Distribution of Medication Possession Ratio.

The distribution of MPRs shows two peaks, one close to zero and one close to one (Fig 3). For not normally distributed outcomes the mean should not be used, but in order to compare with other studies the mean MPR for all medication profiles was calculated to be 0.5498 (SD±0.36). This result indicates that patients were covered with medication for 55% of the observation period on average The respective mean MPRs for the four individual DMD groups were as follows: Avonex 0.5626 (SD±0.37), Betaferon 0.5536 (SD±0.36), Rebif0.5484 (SD±0.36), and Copaxone0.5347 (SD±0.36).

**Fig 4:**
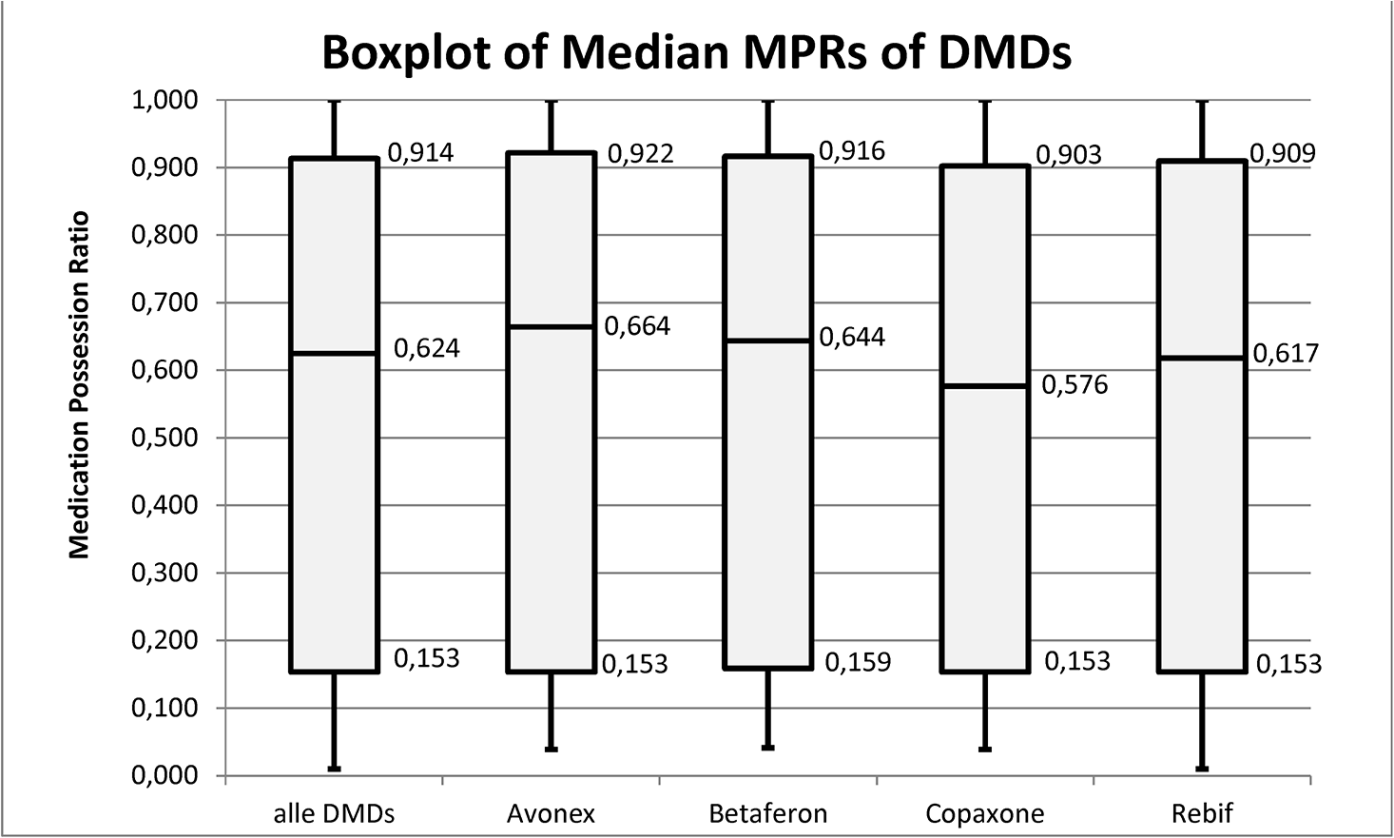
Median Medication Possession Ratios of the four DMD groups.

The median of all MPRs was 0.624. Hence, for half of the profiles patients received medication supplies for less than 62.4% of the follow-up period. The median MPRs for the four study groups were as follows: Avonex 0.664 Betaferon 0.644, Rebif 0.617 and Copaxone 0.576 (Fig 4).

**Fig 5:**
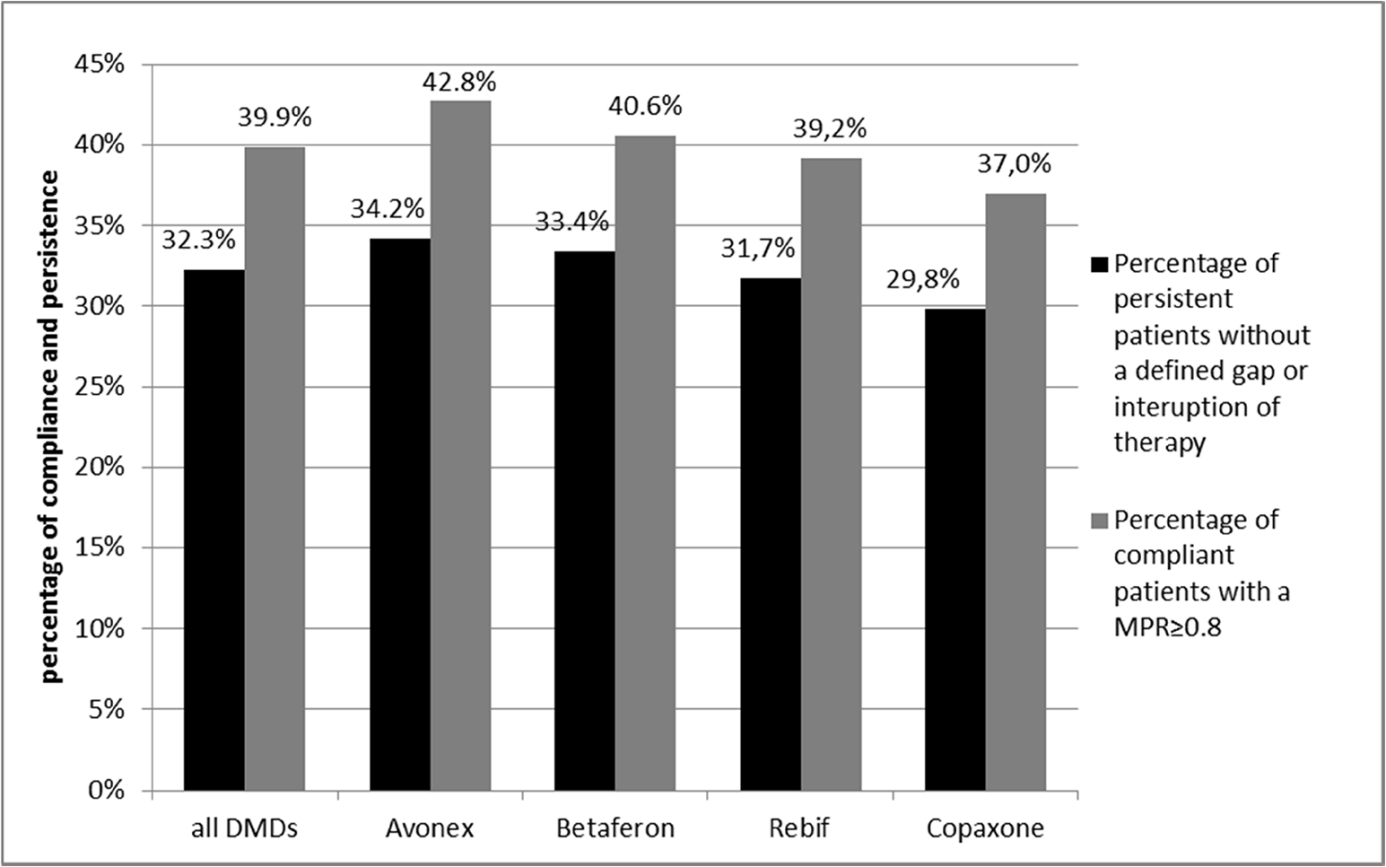
Main analysis: Overview of compliance and persistence in the DMD groups over an observation period of 24 months.

We dichotomized the MPRs by defining MPRs ≥0.8 as compliant and MPRs < 0.8 as non-compliant. The proportion of medication profiles with a MPR ≥ 0.8 among all DMDs was 39.9%. The respective proportions for the four DMD groups were: Avonex 42.8%, Betaferon40.6%, Rebif39.2%, and Copaxone37% (Fig 5).

### Persistence

**Fig 6:**
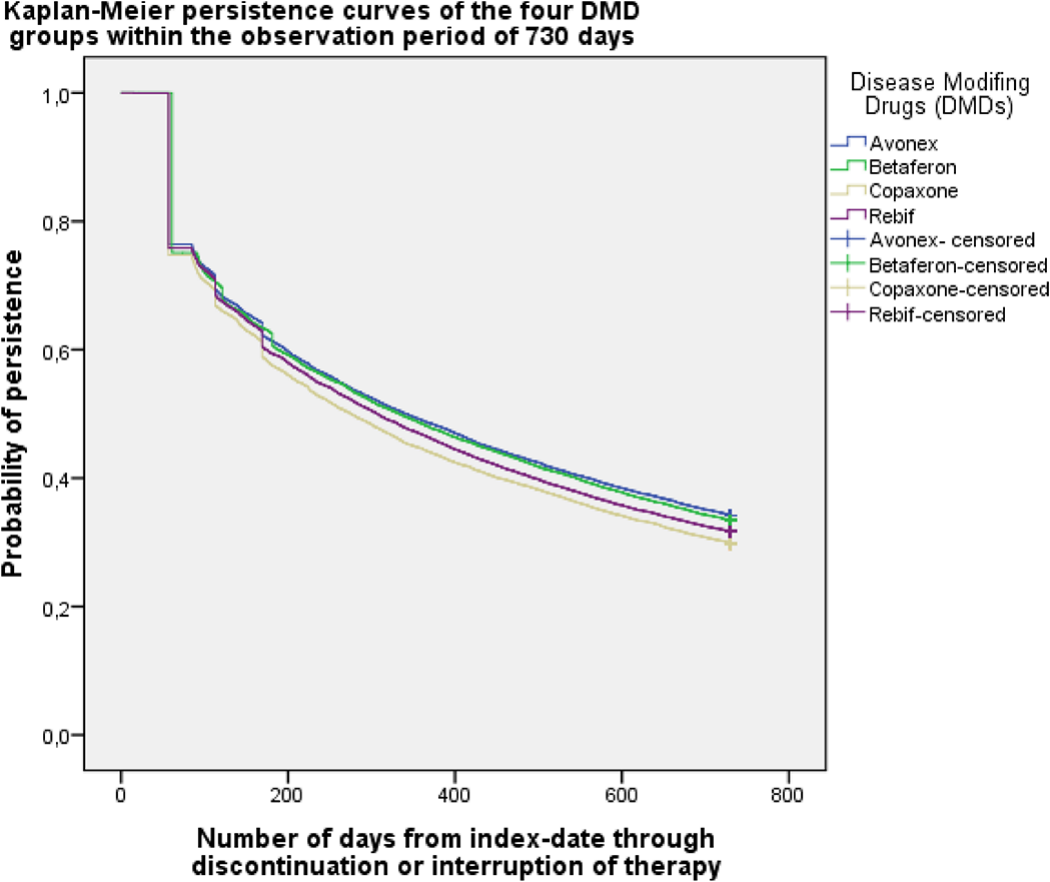
Main analysis: Kaplan-Meier persistence curves of the 4 DMD groups within the observation period of 730 days. First day of non-persistence in relation to the index date.

Over the entire observation period of 24 months, for a total of 32.3% of all included medication profiles patients were considered persistent. Results for the four DMD groups were: Avonex 34.2%, Betaferon33.4%, Rebif31.7%, and Copaxone29.8% (Fig 5). We found that 25% of the initiated therapies cycles were interrupted within 86 days and 50% within 312 days (Fig 6). For 22.6% of all therapy cycles the patient did not receive a second prescription within 180 days after index prescription.

### Sensitivity analyses

To assess the influence of adherence and persistence definitions, we performed three sensitivity analyses.

First, we modified the thresholds for MPR: non-compliance was defined as MPR< 0.7 or MPR< 0.9. Because of the bimodal distribution of the MPR the threshold for the definition of compliance has a comparatively large impact on the result of the overall compliance (Table 2). The closer the threshold comes to 100%, the more the proportion of therapy cycles during which a patient was compliant (allover MPR≥0.70 = 46.1%, MPR≥0.80 = 39.9%, MPR≥0.90 = 28.0%) decreases. In this sensitivity analysis he relative compliance differences between the four DMD groups remained.

**Table 2:** Sensitivity analysis (S1): Proportion of profiles of compliant patients for three different thresholds of MPR.

	MPR≥0.70	MPR≥0.80	MPR≥0.90
All DMDs	46.1%	39.9%	28.0%
Avonex	48.2%	42.8%	30.8%
Betaferon	47.1%	40.6%	28.7%
Rebif	45.6%	39.2%	27.2%
Copaxone	43.5%	37.0%	25.0%

In a second sensitivity analysis we modified the definition of persistence by decreasing the gap in the medication profile to half-fold or increasing the gap to two-fold of the value of the main analysis. For example for a previous prescription with medication supply for 84 days we detected a gap after 84 days plus 42 days (126 days) or 84 days plus 2*84 days (252 days) (Table 3). The proportion of therapy cycles during which a patient was persistent increased by a greater tolerable gap. Overall persistence using a 0.5-fold gap was 19.7%, using a one-fold gap 32.3% and using a two-fold gap 42.5%. The differences in persistence between the four DMD groups once again remained.

**Table 3:** Sensitivity analysis (S2): Proportion of profiles of persistent patients for three different definitions for the tolerable gap in medication profiles.

Persistence	Half-fold duration of medication supply of the previous prescription	One-fold duration of medication supply of the previous prescription	Two-fold duration of medication supply of the previous prescription
All DMDs	19.7%	32.3%	42.5%
Avonex	21.2%	34.2%	44.1%
Betaferon	20.3%	33.4%	43.7%
Rebif	19.1%	31.7%	42.3%
Copaxone	18.2%	29.8%	39.9%

The third sensitivity analysis included only the patients' first medication profile (N = 50,057). Thus, only 4.7% of all medication profiles of the main study cohort were excluded. The influence of undergoing more than one therapy cycles resulted in a decrease in adherence, however, this influence was small (results not shown).

## Discussion

According to the guidelines of scientific associations, the treatment of MS with DMDs has to continue over a long term to gain benefits [30,2]. This study shows that less than 40% of the patients were compliant according to widely accepted definition, i.e., receiving medication for a minimum of 80% of the therapeutically relevant observation period of 24 months. Furthermore, the study revealed that at the end of the observation period only 32.3% of the patients were persistent in terms of having no interruption or having no greater gap in their medication coverage than the one-fold duration of medication supply of the previous prescription. A quarter of the cohort had already discontinued DMD therapy after 86 days. These results are comparable with other studies [31,3,8,10,11,19,22]. Compliance data are not easy to compare because of the different measurement methods, different data sources, and different lengths of observation periods. Studies based on questionnaires seem to result in greater adherence than ones based on claims data [5,7,23], which may be due to social desirability, i.e. patients having a tendency to overestimate their adherence behavior and accordingly to report adherence levels, which are too optimistic.

The bimodal distribution of MPR might be caused by the different reasons why patients stop therapy as found in previous studies [8,24]. They could show that in the first period after the first injection patients stop because of side-effects such like injection-site reactions, flu-like symptoms, headache, for fear of self- injection. Later, stopping therapy is associated with perceived lack of efficacy of the treatment for example because of progress in disease even though they were adherent for a certain period of time.

Remarkably, all four DMDs are equally distributed even though three of them are interferons and one is glatiramer acetate. A 50/50 distribution may be expected. Between 2002 and 2006, there was an overall increase of index prescriptions from 9,458 to 11,128. The three interferons emerged on the German market in 1998, respectively 1999. Approval for Copaxone in the EU was obtained in 2001. It usually takes time to implement new therapies. It appears less likely that prevalence of MS has increased in such a short time period. One may speculate whether the awareness of new disease-modyfiying drugs has led to higher diagnostic rates.

Adherence and persistence were also examined for the four DMD groups, mainly using descriptive methods, because information on possibly predictive covariates such as age, gender, socioeconomic status, side effects, progression stage of disease, duration of disease etc. are not available in the database. Thus it is uncertain whether differences in adherence and persistence between the four study groups are due to the DMDs themselves. Avonex with a compliance of 34.2% and persistence of 42.8% had slightly higher figures than Betaferon with a compliance of 33.4% and persistence of 40.6%, followed by Rebif with a compliance of 31.7% and persistence of 39.2%, and finally Copaxone with a compliance of 29.8% and persistence of 37.0%. Other studies found different orders in levels of adherence, but also suggested that Avonex had the highest level of adherence [5,6,7,10,15,19,21,23,25,31]. One of the deficiencies of the present research was the impossibility of determining the reasons for low adherence levels as well as adherence differences between the four different DMD drug products. It is assumed that the frequency of application of a specific DMD (once a week, three times a week, daily), and the side effect profiles of medications play a role [32].

As WHO reported already in 2003, adherence is a huge challenge in all chronic diseases worldwide. Depending from measurement method, adherence among patients with asthma ranges from 30% to 70% [4]. Grimmsmann, Himmel and Hasford et al. found even poorer adherence among patients in Germany with hypertension [33,34]. Enting et al. found in their research that the proportions of patients adherent to “around the clock” analgesics varied from 59% to 91% [35]. Health care providers and patients should find strategies to improve adherence to distribute rare resources more effective.

One of the strengths of this study was the use of a large sample size.

## Limitations

If a patient had a prescription for DMDs, we assumed MS diagnosed, but we had no data to confirm this assumption. In Germany, DMDs are approved only for MS. As there is no useful off-label use for DMDs, we assumed that a patient who received a DMD had a MS diagnosis. A cessation of therapy in our data might have meant that the patient stopped therapy because of non-adherence. Cessation also could have meant one of the following possibilities: the patient moved away from the region that is covered by the DAPI database; the patient received escalation therapy (2.1% of the cases); the physician decided for any other reason to stop therapy; or the patient became pregnant. Results calculated from claims data can only estimate compliance based on dispensing the medication to the patient. However, no information was available as to whether the prescribed medication was actually applied. The assumption that the patient has complied with a prescribed medication may be open to question. For example, Grymonpre et al. compared compliance derived from patient diaries (94.8% of patients were compliant), tablet counts (65.1% of patients were compliant) and using claims data (89.1% of patients were compliant) and thus found big differences in compliance [36]. Secondary data analyses might be just one method among others like medical event monitoring, self-report, diaries which are all reasonable methods to describe adherence [37].
